# HD-tDCS mitigates the executive vigilance decrement only under high cognitive demands

**DOI:** 10.1038/s41598-024-57917-y

**Published:** 2024-04-03

**Authors:** Klara Hemmerich, Juan Lupiáñez, Elisa Martín-Arévalo

**Affiliations:** Department of Experimental Psychology, and Mind, Brain, and Behavior Research Center (CIMCYC), University of Granada, Campus de Cartuja, s/n, 18071 Granada, Spain

**Keywords:** Attention, Human behaviour

## Abstract

Maintaining vigilance is essential for many everyday tasks, but over time, our ability to sustain it inevitably decreases, potentially entailing severe consequences. High-definition transcranial direct current stimulation (HD-tDCS) has proven to be useful for studying and improving vigilance. This study explores if/how cognitive load affects the mitigatory effects of HD-tDCS on the vigilance decrement. Participants (*N* = 120) completed a modified ANTI-Vea task (single or dual load) while receiving either sham or anodal HD-tDCS over the right posterior parietal cortex (rPPC). This data was compared with data from prior studies (*N* = 120), where participants completed the standard ANTI-Vea task (triple load task), combined with the same HD-tDCS protocol. Against our hypotheses, both the single and dual load conditions showed a significant executive vigilance (EV) decrement, which was not affected by the application of rPPC HD-tDCS. On the contrary, the most cognitively demanding task (triple task) showed the greatest EV decrement; importantly, it was also with the triple task that a significant mitigatory effect of the HD-tDCS intervention was observed. The present study contributes to a more nuanced understanding of the specific effects of HD-tDCS on the vigilance decrement considering cognitive demands. This can ultimately contribute to reconciling heterogeneous effects observed in past research and fine-tuning its future clinical application.

Transcranial direct current stimulation (tDCS) provides the possibility to modulate cortical excitability of specific brain regions^1,2^, which can potentially modify a broad range of cognitive functions^3–6^, including attentional functioning^7^. Applying tDCS to improve and/or maintain performance gains special relevance in contexts where the targeted function is central to a broad range of tasks and degrades quickly over time. This is the case of vigilance, which requires sustaining the focus of attention over long time periods, and remaining alert to detect specific yet unpredictable stimuli^8^. Using tDCS to mitigate this inevitable decrement of vigilance over time has proven to serve as a fruitful intervention^9–12^. Specifically, anodal high-definition (HD) tDCS over the right posterior parietal cortex (rPPC)^13,14^ has shown to mitigate the decrement of executive vigilance (EV), understood as the ability to monitor and execute a specific response to infrequent but relevant stimuli^15^. Whereas it has shown no effect in mitigating the decrement in arousal vigilance (AV), understood as the ability to maintain a basic state of activation that allows responding to any stimuli of the environment in a fast and relatively automatic manner^15^.

A lateralization of sustained attention processes towards the right hemisphere has been established in neuroimaging studies^16–19^, as well as through lesion studies^20,21^. More specifically, lesion studies have identified the rPPC as a hub for spatial attention as well as vigilance^22^, whereas, on a functional level, the rPPC shows a heightened hemodynamic response to infrequently presented targets^17^, maintaining current task goals active as well as responding to (internal or external) novel stimuli^23^. This has led to considering the rPPC as a “convergence node” between the ventral attentional network and the default mode network (DMN), more associated with self-generated thoughts or mind-wandering^24^. Furthermore, imaging data from healthy participants suggests that the superior and inferior parietal cortices (constituting the rPPC) are densely interconnected forming a “structural core”^25^ that in turn is highly connected to other neural regions. This positions the rPPC as a highly relevant target for tDCS, given its functional relevance, as well as the potential benefit of tDCS effects spreading through relevant networks^26,27^. Considering the relevance of the rPPC in vigilance processes, the higher spatial precision achieved in the stimulated area by HD-tDCS, as compared to conventional tDCS^28–30^, is of special benefit for more precisely targeting this region.

To understand the underlying mechanisms of the vigilance decrement and its mitigation, one must consider that it may occur due to a complementary or alternative set of causes. Overload theories (resource-depletion hypothesis) assert that the vigilance decrement occurs due to the consumption of attentional resources with time-on-task due to the demanding nature of vigilance tasks^31,32^, with the associated experience of stress^31–34^. Other accounts (underload theories) posit that the underwhelming nature of vigilance tasks, more associated with boredom^35,36^, ultimately leads to a gradually more mindless execution of the task^37,38^. These theories can be tested empirically by manipulating cognitive demands (i.e., the number of simultaneous tasks to perform and therefore, task instructions to hold in working memory). Overload theories pose that increasing task demands would lead to a greater vigilance decrement, which has indeed been observed under normal conditions^39–41^ and found to be accentuated by sleep deprivation^42^. Underload theories, on the other hand, predict that lowering cognitive demands would lead to a less engaged and more mindless performance, steering thoughts away from the task’s goal^43^, producing the vigilance decrement^44^. Further support for underload theories stems from self-reported high mindlessness predicting worse performance in a vigilance task where targets appear with low frequency^45^, reports of task-induced physiological disengagement (i.e., parasympathetic activation and reduced cardiac reactivity)^46^, and activation of DMN structures with time-on-task^47^. Given this disparity of results, Thomson et al. propose the resource-control account, wherein resources are constant, but executive control declines with time-on-task causing the progressive shift of attentional resources from task-related towards task-unrelated thoughts (mind-wandering)^48^. This account considers that other factors than task demand can modulate the vigilance decrement: observing results such as a mitigated vigilance decrement with increased perceptual variability of the task’s target^49^, where higher difficulty demanding more resources is countered by higher engagement, possibly posing a smaller toll on executive control. Among other theories on the vigilance decrement (for a review see: Fortenbaugh et al.^50^), some accounts represent passive fatigue and active fatigue^51^ as two extremes on an inverse U-shaped function^52^ between performance and cognitive load^53^ or arousal^54^. These models incorporate both underload and overload as two extremes, between which we may attain a middle-ground of optimal performance^53,54^. As a case in point, Luna et al. created three load conditions (single task, dual task, and triple task) using the ANTI-Vea task^15,55^ and observed that the single and triple task groups showed a significant EV decrement, which was mitigated in the dual task group^56^. This further reinforced the view that the EV decrement, present with under and over-demand, is mitigated with intermediate cognitive demands.

The current understanding of how cognitive demands affect the vigilance decrement is still unclear given the disparity of findings^39–41,44,46^, and the current lack of models that explain diverging results. This is further obscured by the contradictory findings when using tDCS to modulate these effects^11,57,58^. A better understanding of cognitive load-dependent effects and their interaction with tDCS effects is needed for a better translation of these results towards applied fields. Critically, a more systematic modulation of task demands and stimulation parameters is required in order to define (i) which conditions lead to a greater vigilance decrement, and (ii) critically, under which conditions the vigilance decrement can be mitigated or reduced. The potential impact of these results can branch into (i) providing a small step towards research parameters to follow for understanding and mitigating the vigilance decrement, shedding some light on the currently often contradictory findings, (ii) adapting real-life contexts to optimize performance in human factor applications where the potential negative consequences of the vigilance decrement are greatest (e.g., air traffic control or security screening^59,60^), and (iii) provide the basis for constructing more efficient intervention or rehabilitation strategies for attention deficits such as those encountered in Attention Deficit and Hyperactivity Disorder (ADHD)^61^ or as a sequelae of stroke^62^, with better informed decisions on when to use compensatory strategies (e.g., reduce task demands to adapt to a lower threshold of what would be considered overdemanding) or restitutive approaches (e.g. training program where threshold of overdemand is increased with tDCS) during rehabilitation. In order to obtain a better roadmap for these outlined applications, further replications and, specifically, more systematic manipulations of cognitive load and tDCS is needed, which was the objective of the present study.

## The present study

In the present study, we applied the task manipulations performed by Luna et al., measuring vigilance in a single and dual task^56^, in combination with HD-tDCS over the rPPC, following the same stimulation protocol as Hemmerich et al.^13^. Further comparisons were made with data from the original triple task studies (standard ANTI-Vea, of two previously collected samples^13,14^). This will allow (i) the replication of prior findings of cognitive load-dependent effects on the vigilance decrement^56^, and (ii) further understanding of whether/how these are affected by HD-tDCS. Given the specificity of HD-tDCS on the EV and not the AV effects^13,14^, and the differences in EV decrements depending on cognitive load^56^, we preregistered the following hypotheses (osf.io/9wfbx) regarding behavioural outcomes: (i) we expected a mitigated EV decrement (significantly reduced linear decrement of hits across task blocks in EV trials) in the anodal HD-tDCS group compared to the sham group performing the single load task, replicating the findings from Luna et al.^56^ in the sham group, and expecting the same beneficial effect of HD-tDCS in the anodal group that had been observed under higher cognitive load^13^, (ii) no EV decrement (no linear decrement) in the dual load task, expecting to replicate the findings from Luna et al.^56^, and therefore, no expected differences between stimulation conditions, and (iii) no modulation of AV performance (i.e., linear increment of SD of RT across blocks) in any load or stimulation group (replicating the specificity observed for the stimulation intervention for EV)^13,14^.

## Methods

### Participants

Participants (*N* = 120) were randomly assigned to perform a single or dual version of the ANTI-Vea task while receiving either sham or anodal HD-tDCS. The sample size of 30 participants per experimental condition matched those of prior studies with the standard ANTI-Vea with a priori estimated sample sizes^13,14^. See Table 1 for demographic data. All participants met the safety inclusion criteria for transcranial electrical stimulation (tES)^63,64^ and magnetic resonance imaging (MRI), had normal or corrected-to-normal vision, were right-handed, and had no known neurological or psychiatric conditions. Participants signed an informed consent form and received monetary compensation for their participation (10€/hour). This study was approved by the Ethical Committee of the University of Granada (2442/CEIH/2021 and 1188/CEIH/2020), in accordance with the 1964 Declaration of Helsinki (last update: Brazil, 2013).

**Table 1:** Sample sizes and demographic data for each experimental condition.

Task load	Stimulation group	*N*	Sex	Age
Single task	Anodal HD-tDCS	*n* = 30	21 female	*M* = 22.03, *SD* = 2.80
	Sham HD-tDCS	*n* = 30	19 female	*M* = 24.03, *SD* = 4.13
Dual task	Anodal HD-tDCS	*n* = 30	20 female	*M* = 22.30, *SD* = 4.13
	Sham HD-tDCS	*n* = 30	14 female	*M* = 23.30, *SD* = 3.99
Total sample		*N* = 120	74 female	*M* = 22.92, *SD* = 3.82

No differences between the four groups were observed neither for Sex, *χ*^2^(3, *N* = 120) = 4.09, *p* = 0.252, nor for Age, *F*(3, 116) = 1.76, *p* = 0.158.

## Apparatus and stimuli

### Behavioural measures

**Figure 1:**
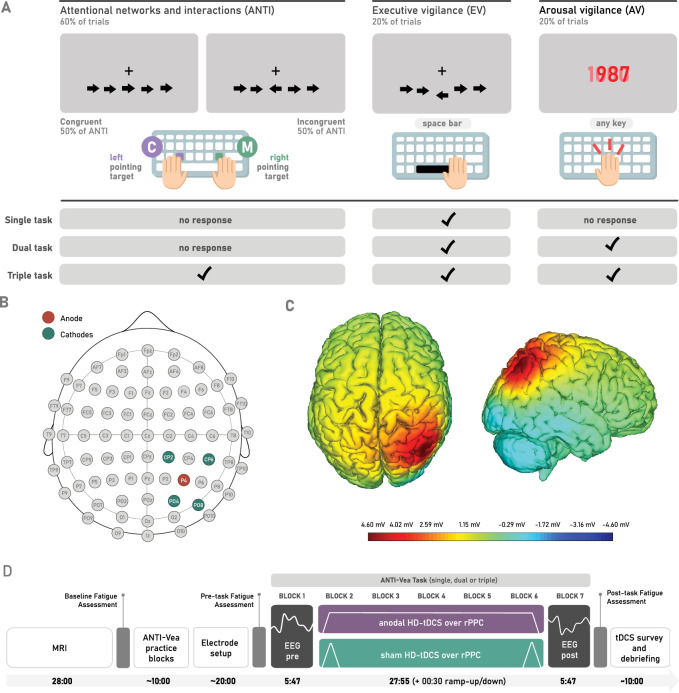
ANTI-Vea Task procedure, electrode setup and resulting E-field simulation, and experimental procedure. (**A**) ANTI, EV, and AV targets of the ANTI-Vea task. The bottom table shows which target(s) participants are instructed to respond to (with a check) for the single, dual, and triple tasks. Note that perceptual load is maintained constant across all task conditions, as only instructions and response coding are modified. Note that both hands are placed over the keyboard at all times, using the left hand to press the “C” key and the right hand for the “M” key, whilst the “spacebar” key and the key chosen by the participant for AV trials can be pressed by any finger/hand (and must thus not be necessarily held constant). (**B**) Electrode setup for HD-tDCS: the anode is placed over P4 (red), and the surrounding cathodes over CP2, CP4, PO4, and PO8 (green), following the same protocol as Hemmerich et al. (**C**) Simulated voltage field obtained from the stimulation protocol from a top and right-hemisphere view. (**D**) Experimental procedure, where the bottom arrow shows the exact or approximate (preceded with a tilde) duration of each step, in minutes. Each fatigue assessment took less than a minute.

Participants performed modified versions of the ANTI-Vea Task (as shown in Fig. 1B), where all trials of the standard task^15^ were presented, but task instructions and responses were coded differently. The ANTI-Vea task is an adapted version of the classical attentional networks task^65^, that includes independent measures of the executive and arousal vigilance components. For this purpose, the task is comprised of three types of trials (ANTI, EV, and AV) that are presented in pseudorandomized order. All ANTI-Vea versions used in this study were run for 7 blocks (560 trials in total). The ANTI trials (60% of total trials) allow measuring the functioning of the classical attentional networks (alerting, orienting, and executive control)^66,67^. These trials present a flanker task where the direction of the target (i.e., a central arrow) must be detected (pressing the *c*-key for left-pointing arrows, and *m*-key for right-pointing arrows) regardless of the direction of the flankers (i.e., surrounding arrows). The EV trials (20% of the total) prompt participants to detect an infrequent and large vertical displacement of the target of the flanker task, by giving an alternative response (pressing the space bar). This sub-task would be akin to signal-detection tasks such as the Mackworth Clock Test (MCT^68^). Lastly, AV trials (remaining 20% of trials) feature a red countdown (instead of the stimuli from ANTI or EV trials), which has to be stopped as fast as possible by pressing any key from the keyboard, akin to the Psychomotor Vigilance Test (PVT^69^). For a more detailed description of the standard task and its parameters, please refer to: Luna et al.^15^, and Luna et al.^55^.

General task instructions across the different load conditions were given for participants to keep their gaze on the fixation point (“ + ”) in the centre of the screen and to respond as fast and as accurately as possible. Then, instructions diverged according to the manipulation of cognitive load, to reflect the correct response for each type of trial as depicted in Fig. 1A. While maintaining perceptual load constant, the manipulation of task instructions and response coding resulted in: (i) a *single task*, which required participants to respond only to EV trials, and (b) a *dual task*, in which participants had to respond to both EV and AV trials. These two groups were then further compared with data from (iii) a *triple task*, where participants had to respond to ANTI, EV, and AV trials (standard ANTI-Vea), collected from two previous studies^13,14^ (*N* = 120).

### HD-tDCS setup

HD-tDCS was applied with a Starstim 8^®^ device and hybrid NG Pistim Electrodes (Ag/AgCl, contact area: 3.14 cm^2^) controlled through NIC v20.6 software (Neuroelectrics^®^, Barcelona). Five of the electrodes, placed in a neoprene headcap, were set up in a 4 × 1 ring-like array, targeting the rPPC by placing the central anode over P4, and the four surrounding cathodes over CP2, CP6, PO4, and PO8 (see Fig. 1B and C). Using a single-blind procedure, anodal (1.5 mA) or sham (0 mA) HD-tDCS was applied according to random group allocation, from the 2nd to the 6th task block (see Fig. 1D). The sham protocol consisted of two ramps (30 s ramp-up and 30 s ramp-down) at protocol onset and offset. The anodal protocol consisted of an initial ramp-up (30 s) followed by active stimulation (~ 28 min), and a ramp-down (30 s) at offset. In this study, electroencephalographic (EEG) signal was recorded during the 1st task block serving as a baseline, and during the 7th block, serving as a post-stimulation measure. Further details regarding this step are beyond the scope of this report as EEG data will not be presented.

### Fatigue assessment

Subjective mental and physical fatigue ratings were assessed throughout the experiment: baseline, pre-task, and post-task (see procedure or Fig. 1D). Responses were recorded through a visual analogue scale: a horizontal line ranging from minimum (left side of the screen) to maximum fatigue (right side). The assessment order for fatigue type was counterbalanced across participants but kept constant for each participant’s session, following the procedure of Luna et al.^56^.

### Procedure

As in Hemmerich et al. the experimental session began with an MRI scan^13^ (~ 28 min), mainly focused on acquiring diffusion-weighted imaging data. This data is being collected as part of a larger research project and will not be covered in the present report. Participants then sat in a separate, dimly lit room to complete the experiment. First, participants completed the baseline fatigue assessment and the ANTI-Vea’s practice blocks (adapted for each load condition). After electrode set-up, participants completed the pre-task fatigue assessment. Then the experimental task started, during which stimulation was applied from the 2nd to the 6th experimental block. Right after the completion of the last (i.e., 7th) experimental block, the post-task fatigue assessment and the tES Survey^70^ were completed.

### Statistical analyses

Following the preregistered plan of analysis, we analysed EV and AV data from baseline (1st block) to the final active or sham stimulation block (6th), following prior HD-tDCS studies^13,14^. Following the standard approach to ANTI-Vea scores^15^, we computed EV indices [Hits (percentage of correct responses), False Alarms (FA), Sensitivity (A’), and Response Bias (B”)] and AV indices [mean RT and standard deviation of RT (SD of RT)]. For EV data, we compared baseline differences in EV indices between stimulation groups using an ANOVA. Then, each index was included in an ANOVA as a dependent variable, with Blocks (1st–6th) as a within-participant factor and Stimulation Group (anodal or sham HD-tDCS) and Task Load (single or dual) as between-participant factors, followed up by partial ANOVAs for each Task Load level. Polynomial contrasts were used to analyse the linear component of each index across Stimulation Group for each Task Load level. Then, the single and dual task data, combined as a *not-triple* condition, were re-analysed jointly with triple-task data^13,14^, combined as a *triple* condition, repeating the above-described analyses (with Updated Task Load) on two balanced samples (*n*_*triple*_ = 120, *n*_*not-triple*_ = 119). Lastly, results for AV data are reported first considering only low-load conditions (i.e., only dual task) and then comparing low and high-load conditions (i.e., dual vs triple task, using data from the present study and data from Hemmerich et al.^13^ to achieve comparable sample sizes in each group).

Note that for all reported ANOVAs, degrees of freedom are reported with Greenhouse–Geisser correction when the sphericity assumption was violated (i.e., p > 0.05 in Mauchly’s test). Additionally, across results, equivalent Bayesian tests are reported to further test the validity of our inferences, as a supplement to non-significant frequentist results. Note Bayes Factors in favour of the null hypothesis (BF_01_) provided for polynomial contrasts on the linear decrement correspond to independent or one sample t-tests completed on the Slope across Blocks (1st–6th).

Methods and Results for Subjective Mental and Physical Fatigue are reported in Appendices E–H of the Supplementary Material.

## Results

### Blinding efficacy

The total amount of self-reported discomfort/sensations associated with stimulation^70^ was significantly different between the Stimulation Groups, *U* = 2190, *p* = 0.037, with higher discomfort reported in the sham (*M* = 2.43, *SD* = 2.08) than in the anodal (*M* = 1.68, *SD* = 1.85) group. This difference seems to be mainly driven by the significantly higher intensity reported for *pinching* in the sham group (*M* = 0.38, *SD* = 0.80) than in the anodal group (*M* = 0.03 *SD* = 0.18), *U* = 2166, *p* = 0.001, without any differences for the remaining sensations (all *p*’s > 0.136, see Appendix A of the Supplementary Material for further statistical details). The higher discomfort reported in the sham group likely led to a higher estimation of belonging to the active stimulation group in the sham (62%) than in the anodal group (42%). However, the guessed active group allocation was not statistically different between Stimulation Groups, χ^2^(2, *N* = 120) = 4.85, *p* = 0.088. Taken together with the evidence for group differences in total discomfort (BF_10_ = 1.07) and pinching (BF_10_ = 0.93) being anecdotal^71^ at most, leads us to conclude that blinding was still effective in the present study.

### EV decrement under lower cognitive demands: single vs. dual cognitive load conditions

**Figure 2:**
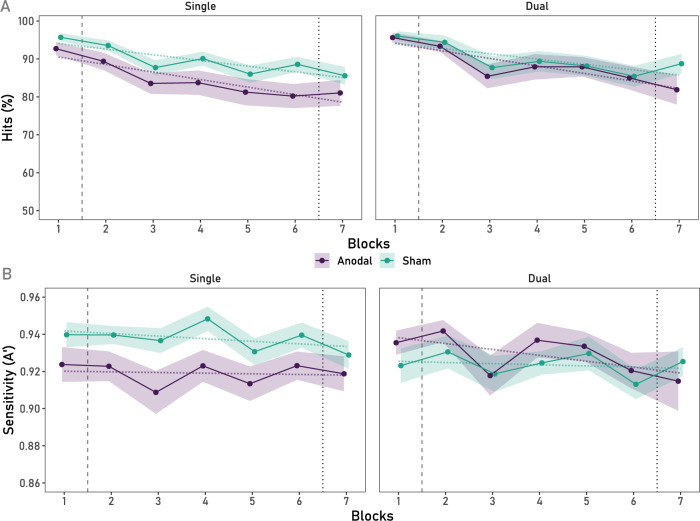
(**A**) Mean % of Hits in EV trials across Blocks for single and dual cognitive load conditions. A linear decrement across Blocks was observed across all conditions. (**B**) Sensitivity (A’) in EV trials across Blocks for the single and dual cognitive load conditions. An effect of Blocks on A’ is observed regardless of the stimulation condition, although the linear component was not significant, whilst the single task condition shows a lower mean A’ (averaged across Blocks) in the anodal compared to the sham condition. *Note.* The dashed vertical line represents the onset of the stimulation protocol. The dotted line represents the offset of the stimulation protocol. The shaded ribbons represent the standard error of the mean (SEM).

Following standard filtering for ANTI-Vea data^55^, outliers (defined based on accuracy < 50% in EV and/or AV trials), excluded one participant (sham-single) from further analyses. There were no significant differences in EV Hits at baseline (Block 1) between the sham and anodal HD-tDCS groups for the single task condition,* F*(1, 57) = 2.07, *p* = 0.156, *ƞ*_*p*_^2^ = 0.04 (BF_01_ = 1.60), or the dual task condition, *F* < 1 (BF_01_ = 3.73). Similarly, no differences between the Stimulation Group at baseline (Block 1) were observed for EV A’ in the single task condition, *F*(1, 57) = 1.92, *p* = 0.172, *ƞ*_*p*_^2^ = 0.03 (BF_01_ = 1.70), or the dual task condition, *F*(1, 58) = 1.20, *p* = 0.278, *ƞ*_*p*_^2^ = 0.02 (BF_01_ = 2.31),

Regarding EV Hits, The Blocks × Stimulation Group × Task Load mixed ANOVA performed on Hits only showed a significant main effect of Blocks, *F*(3.72, 428.18) = 24.27, *p* < 0.001, *ƞ*_*p*_^2^ = 0.17. However, no interactions were significant: Blocks × Stimulation Group, *F* < 1, Blocks × Task Load, *F* < 1, Blocks × Stimulation Group × Task Load, *F* < 1 (all BFs_01_ > 38.27), as shown in Fig. 2A (Note that the reported results span Blocks 1–6, as per our pre-registered plan for analyses. Nonetheless, for clarity, repeating the analyses over Blocks 1–7 yielded the same result. For low-load conditions (single and dual task), the effect of Block remains significant, *F*(4.19, 481.69) = 23.55, *p* < 0.001, *ƞ*_*p*_^2^ = 0.17, without significant interactions (all *F*’s < 1)). A polynomial contrast showed that all groups (joint analysis across experimental conditions) had a significant linear decrement across time, *F*(1, 115) = 51.98, *p* < 0.001, *ƞ*_*p*_^2^ = 0.31. Importantly, in regard to our hypotheses, polynomial contrast showed the expected significant linear decrement of Hits across Blocks in the sham conditions of the single task, *F*(1, 57) = 8.42, *p* = 0.005, *ƞ*_*p*_^2^ = 0.13, which, against our hypotheses was also observed in the sham condition of the dual task, *F*(1, 58) = 12.72, *p* < 0.001, *ƞ*_*p*_^2^ = 0.18. These linear decrements were not significantly different between the two Task Load conditions, *F* < 1 (BF_01_ = 3.25).

Regarding sensitivity (A’) for EV trials, although a main effect of Blocks, *F*(4.34, 499.11) = 2.48, *p* = 0.031, *ƞ*_*p*_^2^ = 0.02, was observed, polynomial contrasts show no significant linear decrement across Blocks (across all conditions), *F*(1, 115) = 1.39, *p* = 0.240, *ƞ*_*p*_^2^ = 0.01 (BF_01_ = 4.89). More importantly, the effect on Blocks did not interact with Stimulation Condition, *F* < 1 (BF_01_ = 127.80), Task Type, *F*(4.34, 499.11) = 1.58, *p* = 0.174, *ƞ*_*p*_^2^ = 0.01 (BF_01_ = 12.90), or an interaction of both *F* < 1 (BF_01_ = 55.47), as depicted in Fig. 2B. As can be observed from Fig. 2B, while the linear decrement is not different across conditions, in the Single Task condition, a difference in overall Hits and A’ can be observed. For mean % Hits (across Blocks 1st–6th) the difference between stimulation conditions did not reach significance, *t*(57) = − 1.88, *p* = 0.065 (BF_01_ = 0.88), whereas a significantly lower mean A’ (across Blocks 1–6) is observed in the sham single task condition (*M* = 0.94, *SD* = 0.03), compared to the anodal single task condition (*M* = 0.92, *SD* = 0.03), *t*(57) = − 2.76,* p* = 0.008. Refer to Appendix B of the Supplementary Material for further results on the remaining EV indices (FA and B”).

### EV decrement under effects of increased cognitive load: single and dual cognitive load conditions vs. triple load

Baseline (i.e., 1st Block) Hits for EV trials were significantly lower for the triple task condition (*M* = 82%, *SD* = 15), compared to the single (*M* = 94%, *SD* = 8) and dual (M = 96%, *SD* = 7) conditions, *F*(2, 234) = 37.60, *p* < 0.001, *ƞ*_*p*_^2^ = *0.24*. However, and importantly, within the triple task condition, there were no significant differences between Stimulation Groups, *F* < 1 (BF_01_ = 3.85). Similarly, no baseline differences were observed for EV A’, *F* < 1 (BF_01_ = 5.01).

**Figure 3:**
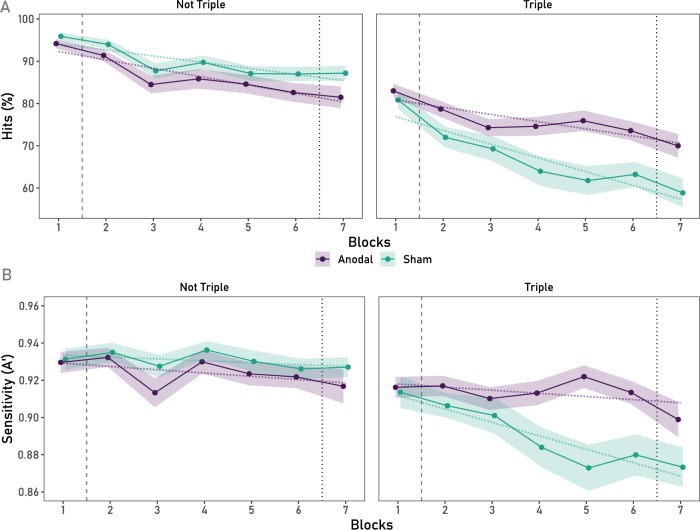
(**A**) Mean % of Hits and in EV trials across blocks combined for the single and dual task conditions (low-load, left), and triple task condition (high-load, right). The above-reported linear decrement in low load conditions, without an effect of HD-tDCS stands in contrast with a significantly lower linear decrement in the anodal compared to the sham HD-tDCS condition of the triple task. (**B**) Sensitivity (A’) in EV trials across Blocks for the low-load condition (left) and the high-load condition (right). In the sham triple task condition, a much steeper decrement of A’ is observed, compared to the non-significant linear component in the triple anodal condition, which is comparable to both low-load conditions. *Note.* The dashed vertical line represents the onset of the stimulation protocol. The dotted line represents the offset of the stimulation protocol. The shaded ribbons represent the SEM.

The ANOVA performed on Hits in EV trials with Blocks as a within participants variable and Stimulation Group and Updated Task Load (triple/not-triple) as between-participant factors, reflected a main effect of Block, *F*(4.31, 513.24) = 21.42, *p* < 0.001, *ƞ*_*p*_^2^ = 0.15, which interacted significantly with Stimulation Group, *F*(4.31, 513.24) = 3.69, *p* = 0.005, *ƞ*_*p*_^2^ = 0.03. Importantly, the three-way Blocks × Stimulation Group × Updated Task Load interaction was significant, *F*(4.24, 999.51) = 2.97, *p* = 0.017, *ƞ*_*p*_^2^ = 0.01 (For transparency, to complement the pre-registered analyses over Blocks 1–6, repeating the same analyses across Blocks 1–7, yields the same results: the main effect of Block, *F*(5.01, 1181.07) = 44.78, *p* < 0.001, *ƞ*_*p*_^2^ = 0.16, and the critical three-way Block × Stim × Updated Task Type interaction remain significant, *F*(5.01, 1181.07) = 2.91, *p* = 0.013, *ƞ*_*p*_^2^ = 0.01). Polynomial contrasts completed on the grouped (triple vs. not-triple) data showed that the linear decrement between the anodal and sham conditions was not different for the not-triple condition, *F* < 1 (BF_01_ = 4.09), whereas it was for the triple task condition, *F*(1, 119) = 8.62, *p* = 0.004, *ƞ*_*p*_^2^ = 0.07. Bayesian analyses further showed that there was moderate evidence (BF_10_ = 5.66) for this mitigated EV decrement in the triple task anodal group, as can be seen in Fig. 3 (right), compared to extreme evidence (BF_01_ = 145.25) against a significant interaction in the not-triple task condition, as shown in Fig. 3 (left). Lastly, there was a significant difference in the linear decrement observed between sham conditions between the not-triple and triple tasks, *F*(1, 117) = 7.99, *p* = 0.006, *ƞ*_*p*_^2^ = 0.06, reflecting, the significantly greater EV decrement under high compared to lower load conditions. In contrast, the anodal not-triple and triple conditions’ linear decrement were not significantly different from each other, *F*(1, 119) = 1.02, *p* = 0.316, *ƞ*_*p*_^2^ = 0.01 (BF_01_ = 3.17), which indicates that HD-tDCS in the triple task conditions seems to mitigate the vigilance decrement up to the performance level observed for the single or dual task conditions.

Notably, Sensitivity (A’) also decreased significantly across Blocks, *F*(4.59, 1084) = 3.82, *p* = 0.003, *ƞ*_*p*_^2^ = 0.02, and was modulated by Stimulation Condition, *F*(4.59, 1084) = 2.72, *p* = 0.022, *ƞ*_*p*_^2^ = 0.01, but not by Updated Task Load, *F*(4.59, 1084) = 2.05, *p* = 0.08, *ƞ*_*p*_^2^ = 0.01 (BF_01_ = 16.71). Importantly, the triple interaction was significant, *F*(4.59, 1084) = 2.82, *p* = 0.019, *ƞ*_*p*_^2^ = 0.01. Polynomial contrasts reflected a significant linear decrement in A’ in the triple task sham group, *F*(1, 119) = 23.36, *p* < 0.001, *ƞ*_*p*_^2^ = 0.16, significantly different from the linear decrement in the triple task anodal group, *F*(1, 119) = 12.11, *p* < 0.001, *ƞ*_*p*_^2^ = 0.09, where, notably, no significant linear decrement was observed, *F* < 1 (BF_01_ = 7.01), as can be seen in Fig. 3 (bottom panel, right). See Appendix C of the Supplementary Material for further results on the remaining indices for EV trials (FA and B”).

### AV decrement: dual vs. triple load conditions

**Figure 4:**
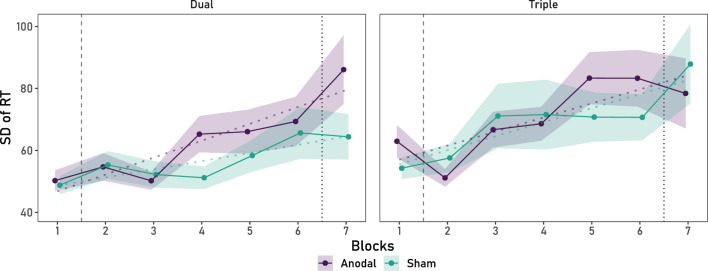
AV decrement (increment of SD of RT with time-on-task) as a function of stimulation condition for the dual task (left) and the triplet ask condition (right). No differences between the linear increment of SD of RT across Blocks were observed between Stimulation Groups of either task condition. *Note.* The dashed vertical line represents the onset of the stimulation protocol. The dotted line represents the offset of the stimulation protocol. The shaded ribbons represent the SEM.

For the dual task AV data there were no significant baseline differences between the two Stimulation Groups on SD of RT, *F* < 1 (BF_01_ = 3.64). As predicted, there was a significant AV decrement, shown as an increment in the SD of RTs to AV trials across Blocks, *F*(3.39, 196.87) = 4.86, *p* = 0.002, *ƞ*_*p*_^2^ = 0.08, which was not modulated by HD-tDCS, *F* < 1 (BF_01_ = 16.26) (To complement the pre-registered analyses over Blocks 1–6, if the same analyses are repeated over Blocks 1–7, the same results are observed: comparing the AV (SD of RT) across the dual and triple tasks also showed a significant effect of Blocks, Block: *F*(4.11, 476.88) = 11.23, *p* < 0.001, *ƞ*_*p*_^2^ = 0.09, but no significant interactions (*p*’s ≥ 0.145)). Polynomial contrasts further showed that whilst there was no significant linear increment in the sham group, *F*(1, 58) = 3.23, *p* = 0.077, *ƞ*_*p*_^2^ = 0.05 (BF_01_ = 0.54), it was significant for the anodal group, *F*(1, 58) = 7.90, *p* = 0.007, *ƞ*_*p*_^2^ = 0.12. Importantly, the linear increment was not significantly different between Stimulation Groups, *F* < 1 (BF_01_ = 3.07), as shown in Fig. 4.

Finally, an ANOVA performed on SD of RT, contrasting the dual and triple conditions, showed a significant AV decrement (increment of SD of RT) across Blocks, *F*(4.02, 466.80) = 9.32, *p* < 0.001, *ƞ*_*p*_^2^ = 0.07. However, this did not interact with Stimulation Condition, *F*(4.02, 466.80) = 1.35, *p* = 0.249, *ƞ*_*p*_^2^ = 0.071 (BF_01_ = 21.70), or Task Load, *F*(4.02, 466.80) = 11.62, *p* = 0.167, *ƞ*_*p*_^2^ = 0.01 (BF_01_ = 12.10), nor was there a significant triple interaction, *F* < 1 (BF_01_ = 15.09), as can be seen in Fig. 4. Refer to Appendix D of the Supplementary Material for further AV results (Mean RT in AV trials).

## Discussion

This study aimed at investigating the influence of cognitive load and HD-tDCS, as well as their interaction, on the EV decrement. To this end, we manipulated task load (single or dual) and HD-tDCS application over the rPPC (sham vs. active). Contrary to our preregistered hypotheses, we observed no differences between the EV decrement in the single and dual task conditions and no modulation of this decrement by HD-tDCS. As expected, neither cognitive load nor HD-tDCS modulated the AV decrement. Importantly, when contrasted with prior results using a triple task, we are able to expand evidence on the specific effect of rPPC HD-tDCS on the executive component of vigilance^13,14^: the mitigatory effect of HD-tDCS is only evident under conditions of high cognitive demand.

Against our pre-registered hypothesis, we did not replicate the findings of Luna et al.^56^, as the single and dual load conditions both showed a significant EV decrement with time-on-task, without any differences across load conditions. Some studies report similar null effects comparing single and dual tasks^32,72^, or no vigilance decrement at all regardless of the load condition^73,74^. However, most of the literature is either skewed towards underload (observing larger decrements with lower task demands^44^ or higher engagement^75^) or overload theories (observing greater vigilance decrements with increased task demands by adding a secondary task^39–41^ or increasing instruction complexity^72^), without any clear consensus. One possible explanation for our diverging results is that single and dual tasks yielded conditions that were qualitatively not sufficiently different and therefore processed similarly. Under these low to medium load conditions, available resources may suffice to (somewhat successfully) complete the task and mind-winder in parallel (maintaining the same level of performance across slightly differing demand conditions). This could be explained by the *resource-control account*, as executive control decreases with time-on-task, gradually tipping the balance from task-related towards task-unrelated thoughts^48^. The single and dual tasks may operate at a relatively low “tipping point”. Importantly, the EV decrement has been recently linked with the loss of executive control with time-on-task in the standard ANTI-Vea (triple task)^76^. Future research systematically manipulating task demands in a within-participants design could explore: (i) whether executive control measures and the EV decrement are related when task demands are reduced, and (ii) how each load level influences the presence of task-unrelated thoughts.

Contrary to the expected mitigated EV decrement in the single group receiving active HD-tDCS and no effect of HD-tDCS on EV performance in the dual group, we observed no mitigatory –or detrimental– effect of stimulation in either the single or dual task condition. Similar results have been observed with the Sustained Attention to Response Task (SART) comparable to our single task condition: prefrontal tDCS did not affect target accuracy^57^, and anodal or cathodal tDCS over the right inferior parietal cortex (rIPL) did not affect error rates or RTs^77^. Similarly, another study reports null effects of anodal tDCS over the left PFC in a dual working memory task^58^. However, there are also some reports of detrimental effects of higher doses of both anodal and cathodal tDCS over the rIPL on accuracy in the SART^78^, and beneficial effects on accuracy with anodal HD-tDCS over the left dorsolateral prefrontal cortex (DLPFC) regardless of the task demand condition of a standard and a modified SART^79^. Lastly, it has been suggested that prefrontal tDCS may modulate sustained attention by affecting its higher-order sub-processes, rather than simple target detection^7^, which could partially explain the absence of effects of tDCS in low demanding conditions.

**Figure 5:**
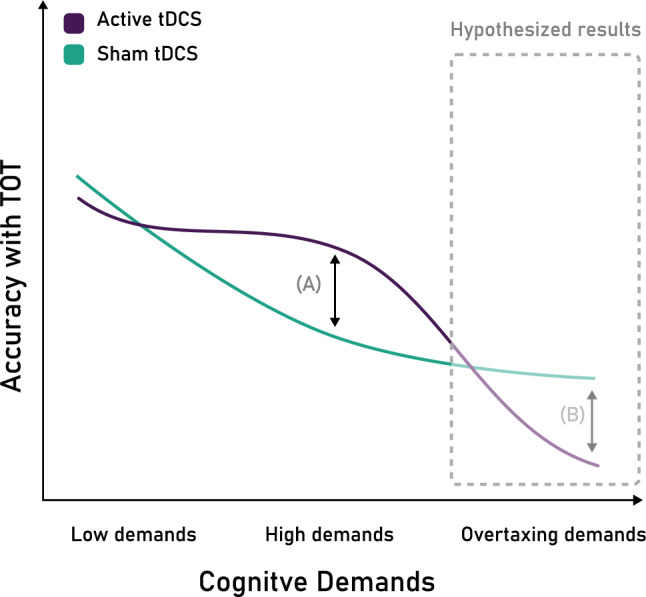
Observed and hypothesized interaction of cognitive demands and HD-tDCS over rPPC on the accuracy performance with time-on-task (TOT), with lower values depicting a greater EV decrement. (**A**) Beneficial effect of active HD-tDCS over the rPPC, mitigating the EV decrement, as observed in the present study. (**B**) Further increasing task demands to a level that is overtaxing, would potentially lead to even worse EV performance, which could be further deteriorated by the application of active tDCS –as conceptualized and observed by Roe et al.^11^.

In contrast to the null effect of HD-tDCS on the EV decrement in the low and medium load conditions, the mitigatory effect of rPPC HD-tDCS was only observed in the most demanding condition (triple task). The EV decrement in the sham triple-task condition was more pronounced than under single and dual load, which was mitigated in the HD-tDCS condition. Similar results have been observed with anodal tDCS over the right DLPFC, leading to improved accuracy under the highest load condition of a working memory task^80^, and anodal tDCS over the left DLPFC leading to delayed beneficial effects on multitasking but not on single task performance^81^. Other studies also suggest that tDCS over right prefrontal or parietal areas can lead to detrimental effects on task performance under objective^11^ and subject-specific high load conditions^82^. In contrast, some studies have reported beneficial effects of cathodal tDCS for maintaining or improving performance in high load conditions^83,84^. Studies on the intersection of cognitive load and tDCS are still rather scarce and yield no clear conclusions. While the inconsistencies across the existing literature are partially explained by the variability between stimulation procedures, cognitive processes studied, and tasks used across these different studies, a crucial factor to consider is the conceptualization of cognitive load and how its levels are established. Roe et al. argue that *“[…] using a load level that overtaxes cognitive capacity, as well as making use of a wider range of load levels (i.e., more than two), is preferable if one’s goal is to investigate the interaction between tDCS and cognitive load*”^11^. Precisely, the high load condition of our study, although complex and demanding, is not overtaxing, as was the case for the high load condition of studies reporting detrimental effects of anodal tDCS^11,82^. The effects of tDCS on the vigilance decrement are likely to depend less on the externally imposed and conceptualized levels of cognitive load, but rather on the specific demand they impose on each individual, and the specific neural state they induce^85^. Therefore, as illustrated in Fig. 5, high but manageable cognitive demands could lead to beneficial effects of anodal tDCS, as observed in the present study, where increasing neural excitability may further excite task-relevant processes. However, we hypothesize that when further increasing demands to a level where task performance cannot be maintained, the effects of anodal tDCS would be detrimental, as increasing the excitability of overtaxed neural circuits is likely to disrupt task performance. This might also explain facilitatory effects of cathodal tDCS in tasks with high demand^83,84^, where inhibitory processes could reduce over-demand. Lastly, in the lower load conditions (single and dual task), a ceiling effect of the modulatory effects of HD-tDCS may be taking place.

Another relevant result of the present study is the finding that performance gains, namely, the improved accuracy in target detection for EV trials, were due to improved sensitivity (i.e., ability to discriminate signal from noise), and not due to shifts in the response bias (i.e., the adoption of a more liberal response criterion, which would merely increase hits at the cost of increasing false alarms). While some studies do report similar results^86,87^, signal detection theory measures are not discussed in most studies exploring the effect of tDCS on vigilance, and opposite findings have also been reported showing greater sensitivity declines in less demanding tasks^88^. Thus, whilst requiring further replication, for now, our results highlight that when HD-tDCS mitigates the EV decrement (in high demand conditions), it does so by improving performance in a precise manner.

Taken together, our results further point to the fact that underlying mechanisms driving EV performance are not being properly explored with the tools at hand. As suggested above, a better understanding of what is causing the vigilance decrement, as would do, for example, collecting thought probes throughout the task, would help further understand the present results. Although future challenges still lie in the fact that the presence of mind-wandering is not a fool-proof sign of underload, as the presence of mind-wandering does not always predict performance costs^89^, nor does the manipulation of task demands always lead to different mind-wandering rates^79^. Future research could bridge this gap by including, not only thought-probes in vigilance tasks but also including other more objective measures of engagement, such as eye movements^90^. Finally, given that the vigilance decrement can be shaped by a myriad of different factors^91^, future research should refine their approach in studying cognitive load dependent effects on vigilance, in which considering individual differences should be a key factor.

However, despite the above-mentioned limitations and open questions, the present findings can tentatively inform future decisions in research and clinical settings. The cognitive-load dependent effects of HD-tDCS on the EV decrement as observed in the present study underline the importance of considering cognitive load as an essential factor in: (i) predicting stimulation outcomes, and (ii) tailoring the interactions of demands and tDCS individually. Regarding the first point, whilst future research is needed to understand the generalizability of these results, our data suggests that in areas where a tDCS intervention is to be applied but cognitive demands cannot be modified or adapted, a prediction (based on behavioural data) could be made as of how successful a tDCS intervention would actually be. If the task is overdemanding, the intervention is likely to not adequately induce plastic changes towards the desired outcomes, whereas, if the task is under-demanding, a ceiling effect might hamper any real efficacy of the stimulation as well. While prior to such applications, further research would be needed, this consideration could be a first step in more precisely delineating the intervention and, potentially, offer a broad guideline that could avoid devoting resources to null findings. Regarding the second point, when the cognitive demands can be individually assessed and adjusted to an optimal level, the efficacy of interventions focused on the rehabilitation of attentional functions could be greatly improved. In a clinical setting, attention deficits such as those elicited by ADHD^61^ or as a sequelae of a stroke^62^, could lead to the subjective and individual experience of high cognitive demands or even result in an over-taxing of resources in context that are considered to be of low demand under normal circumstances. Given that the threshold of what is considered overdemanding is not even uniform among healthy participants^58,82^, it will likely be even more heterogenous in these clinical populations. Therefore, instead of externally imposing a fixed demand, individually tailoring demand levels of cognitive training tasks to individual capacity^58,82^ and gradually increasing task demands, for online use in a tDCS intervention may ensure that the neuroplastic effect of tDCS actually reinforces effective task-resolution and learning processes^85^ as a restitutive approach to regain attentional functioning.

## Conclusions

According to our results, the EV decrement does not seem to be modulated by cognitive load under relatively undemanding conditions (towards improved performance in the dual load group, as was reported by Luna et al.^56^). Indeed, both single and dual load conditions showed a similar vigilance decrement across time. Under these conditions (single and dual cognitive load), additionally, HD-tDCS does not affect EV performance. However, under conditions with higher demand (i.e., triple task) there is a steeper vigilance decrement compared to lower load conditions, which was mitigated via anodal HD-tDCS over the rPPC. This study highlights the fact that task demands should be an important factor in considering the efficacy of a tDCS intervention on vigilance performance. This will allow a better understanding of the vigilance decrement in itself and facilitate a more effective translation of these results into clinical settings.

### Supplementary Information

Supplementary Information.

## Data Availability

The data that support the findings of this study are openly available in the Open Science Framework (OSF) at https://osf.io/876fe/. Materials of the study can be found here: https://osf.io/wef3q/.

## References

[1] Nitsche MA (2008). Transcranial direct current stimulation: State of the art 2008. Brain Stimulat..

[2] Liu A (2018). Immediate neurophysiological effects of transcranial electrical stimulation. Nat. Commun..

[3] Coffman BA, Clark VP, Parasuraman R (2014). Battery powered thought: Enhancement of attention, learning, and memory in healthy adults using transcranial direct current stimulation. NeuroImage.

[4] Antal A (2022). Non-invasive brain stimulation and neuroenhancement. Clin. Neurophysiol. Pract..

[5] Davis SE, Smith GA (2019). Transcranial direct current stimulation use in warfighting: Benefits, risks, and future prospects. Front. Hum. Neurosci..

[6] Kuo M-F, Nitsche MA (2012). Effects of transcranial electrical stimulation on cognition. Clin. EEG Neurosci..

[7] Reteig LC, Talsma LJ, van Schouwenburg MR, Slagter HA (2017). Transcranial electrical stimulation as a tool to enhance attention. J. Cogn. Enhanc..

[8] Parasurman R, Warm JS, Dember W, Mark LS, Warm JS, Huston RL (1987). Vigilance: Taxonomy and utility. Ergonomics and Human Factors.

[9] Brosnan MB (2018). Prefrontal modulation of visual processing and sustained attention in aging, a tDCS–EEG coregistration approach. J. Cogn. Neurosci..

[10] Dai J (2022). The neuroelectrophysiological and behavioral effects of transcranial direct current stimulation on executive vigilance under a continuous monotonous condition. Front. Neurosci..

[11] Roe JM (2016). The effects of tDCS upon sustained visual attention are dependent on cognitive load. Neuropsychologia.

[12] Roy LB, Sparing R, Fink GR, Hesse MD (2015). Modulation of attention functions by anodal tDCS on right PPC. Neuropsychologia.

[13] Hemmerich K, Lupiáñez J, Luna FG, Martín-Arévalo E (2023). The mitigation of the executive vigilance decrement via HD-tDCS over the right posterior parietal cortex and its association with neural oscillations. Cereb. Cortex.

[14] Luna FG, Román-Caballero R, Barttfeld P, Lupiáñez J, Martín-Arévalo E (2020). A High-Definition tDCS and EEG study on attention and vigilance: Brain stimulation mitigates the executive but not the arousal vigilance decrement. Neuropsychologia.

[15] Luna FG, Marino J, Roca J, Lupiáñez J (2018). Executive and arousal vigilance decrement in the context of the attentional networks: The ANTI-Vea task. J. Neurosci. Methods.

[16] Pardo JV, Fox PT, Raichle ME (1991). Localization of a human system for sustained attention by positron emission tomography. Nature.

[17] Stevens MC, Calhoun VD, Kiehl KA (2005). Hemispheric differences in hemodynamics elicited by auditory oddball stimuli. NeuroImage.

[18] Langner R, Eickhoff SB (2013). Sustaining attention to simple tasks: A meta-analytic review of the neural mechanisms of vigilant attention. Psychol. Bull..

[19] Lim J (2010). Imaging brain fatigue from sustained mental workload: An ASL perfusion study of the time-on-task effect. NeuroImage.

[20] Koski L, Petrides M (2001). Time-related changes in task performance after lesions restricted to the frontal cortex. Neuropsychologia.

[21] Molenberghs P (2009). Lesion neuroanatomy of the sustained attention to response task. Neuropsychologia.

[22] Malhotra P, Coulthard EJ, Husain M (2009). Role of right posterior parietal cortex in maintaining attention to spatial locations over time. Brain.

[23] Singh-Curry V, Husain M (2009). The functional role of the inferior parietal lobe in the dorsal and ventral stream dichotomy. Neuropsychologia.

[24] Giacometti Giordani L, Crisafulli A, Cantarella G, Avenanti A, Ciaramelli E (2023). The role of posterior parietal cortex and medial prefrontal cortex in distraction and mind-wandering. Neuropsychologia.

[25] Hagmann P (2008). Mapping the structural core of human cerebral cortex. PLoS Biol..

[26] Rosenberg MD (2016). A neuromarker of sustained attention from whole-brain functional connectivity. Nat. Neurosci..

[27] Cosmo C (2015). Spreading effect of tDCS in individuals with attention-deficit/hyperactivity disorder as shown by functional cortical networks: A randomized, double-blind. Sham-Controlled Trial. Front. Psychiatry.

[28] Edwards D (2013). Physiological and modeling evidence for focal transcranial electrical brain stimulation in humans: A basis for high-definition tDCS. NeuroImage.

[29] Alam M, Truong DQ, Khadka N, Bikson M (2016). Spatial and polarity precision of concentric high-definition transcranial direct current stimulation (HD-tDCS). Phys. Med. Biol..

[30] Kuo H-I (2013). Comparing cortical plasticity induced by conventional and high-definition 4 × 1 ring tDCS: A neurophysiological study. Brain Stimulat..

[31] Warm JS, Parasuraman R, Matthews G (2008). Vigilance requires hard mental work and is stressful. Hum. Factors J. Hum. Factors Ergon. Soc..

[32] Grier RA (2003). The vigilance decrement reflects limitations in effortful attention, not mindlessness. Hum. Factors J. Hum. Factors Ergon. Soc..

[33] Szalma JL (2004). Effects of sensory modality and task duration on performance, workload, and stress in sustained attention. Hum. Factors J. Hum. Factors Ergon. Soc..

[34] Dillard MB (2019). Vigilance tasks: Unpleasant, mentally demanding, and stressful even when time flies. Hum. Factors.

[35] Danckert J, Merrifield C (2018). Boredom, sustained attention and the default mode network. Exp. Brain Res..

[36] Yakobi O, Boylan J, Danckert J (2021). Behavioral and electroencephalographic evidence for reduced attentional control and performance monitoring in boredom. Psychophysiology.

[37] Smallwood J, Schooler JW (2006). The restless mind. Psychol. Bull..

[38] Smallwood J, Schooler JW (2015). The science of mind wandering: Empirically navigating the stream of consciousness. Annu. Rev. Psychol..

[39] Epling SL, Russell PN, Helton WS (2016). A new semantic vigilance task: Vigilance decrement, workload, and sensitivity to dual-task costs. Exp. Brain Res..

[40] Head J, Helton WS (2014). Sustained attention failures are primarily due to sustained cognitive load not task monotony. Acta Psychol. (Amst.).

[41] Smit AS, Eling PATM, Coenen AML (2004). Mental effort causes vigilance decrease due to resource depletion. Acta Psychol. (Amst.).

[42] Chua EC-P, Fang E, Gooley JJ (2017). Effects of total sleep deprivation on divided attention performance. PLOS ONE.

[43] Risko EF, Anderson N, Sarwal A, Engelhardt M, Kingstone A (2012). Everyday attention: Variation in mind wandering and memory in a lecture: Mind wandering. Appl. Cogn. Psychol..

[44] Ariga A, Lleras A (2011). Brief and rare mental “breaks” keep you focused: Deactivation and reactivation of task goals preempt vigilance decrements. Cognition.

[45] Manly T (1999). The absent mind: Further investigations of sustained attention to response. Neuropsychologia.

[46] Pattyn N, Neyt X, Henderickx D, Soetens E (2008). Psychophysiological investigation of vigilance decrement: Boredom or cognitive fatigue?. Physiol. Behav..

[47] Salihu AT, Hill KD, Jaberzadeh S (2022). Neural mechanisms underlying state mental fatigue: A systematic review and activation likelihood estimation meta-analysis. Rev. Neurosci..

[48] Thomson DR, Besner D, Smilek D (2015). A resource-control account of sustained attention: Evidence from mind-wandering and vigilance paradigms. Perspect. Psychol. Sci..

[49] Thomson DR, Smilek D, Besner D (2015). Reducing the vigilance decrement: The effects of perceptual variability. Conscious. Cogn..

[50] Fortenbaugh FC, DeGutis J, Esterman M (2017). Recent theoretical, neural, and clinical advances in sustained attention research: Sustained attention research. Ann. N. Y. Acad. Sci..

[51] Saxby DJ, Matthews G, Warm JS, Hitchcock EM, Neubauer C (2013). Active and passive fatigue in simulated driving: Discriminating styles of workload regulation and their safety impacts. J. Exp. Psychol. Appl..

[52] Yerkes RM, Dodson JD (1908). The relation of strength of stimulus to rapidity of habit-formation. J. Comp. Neurol. Psychol..

[53] McWilliams T, Ward N (2021). Underload on the road: Measuring vigilance decrements during partially automated driving. Front. Psychol..

[54] Esterman M, Rothlein D (2019). Models of sustained attention. Curr. Opin. Psychol..

[55] Luna FG, Barttfeld P, Martín-Arévalo E, Lupiáñez J (2021). The ANTI-Vea task: Analyzing the executive and arousal vigilance decrements while measuring the three attentional networks. Psicológica J..

[56] Luna FG, Barttfeld P, Martín-Arévalo E, Lupiáñez J (2022). Cognitive load mitigates the executive but not the arousal vigilance decrement. Conscious. Cogn..

[57] Filmer HL, Griffin A, Dux PE (2019). For a minute there, I lost myself … dosage dependent increases in mind wandering via prefrontal tDCS. Neuropsychologia.

[58] Borragán G (2018). Transcranial direct current stimulation does not counteract cognitive fatigue, but induces sleepiness and an inter-hemispheric shift in brain oxygenation. Front. Psychol..

[59] Yin Z, Rau P-LP, Li Z, Harris D (2019). Impacts of automation reliability and failure modes on operators’ performance in security screening. Engineering Psychology and Cognitive Ergonomics.

[60] Kharoufah H, Murray J, Baxter G, Wild G (2018). A review of human factors causations in commercial air transport accidents and incidents: From to 2000–2016. Prog. Aerosp. Sci..

[61] Pievsky MA, McGrath RE (2018). The neurocognitive profile of attention-deficit/hyperactivity disorder: A review of meta-analyses. Arch. Clin. Neuropsychol..

[62] Brosnan MB (2022). Lost in time: Temporal monitoring elicits clinical decrements in sustained attention post-stroke. J. Int. Neuropsychol. Soc..

[63] Rossi S, Hallett M, Rossini PM, Pascual-Leone A (2009). Safety, ethical considerations, and application guidelines for the use of transcranial magnetic stimulation in clinical practice and research. Clin. Neurophysiol..

[64] Antal A (2017). Low intensity transcranial electric stimulation: Safety, ethical, legal regulatory and application guidelines. Clin. Neurophysiol..

[65] Fan J, Mccandliss BD, Sommer T, Raz A, Posner MI (2002). Testing the efficiency and independence of attentional networks. J. Cogn. Neurosci..

[66] Callejas A, Lupiáñez J, Tudela P (2004). The three attentional networks: On their independence and interactions. Brain Cogn..

[67] Petersen SE, Posner MI (2012). The attention system of the human brain: 20 years after. Annu. Rev. Neurosci..

[68] Mackworth NH (1948). The breakdown of vigilance during prolonged visual search. Q. J. Exp. Psychol..

[69] Lim J, Dinges DF (2008). Sleep deprivation and vigilant attention. Ann. N. Y. Acad. Sci..

[70] Fertonani A, Ferrari C, Miniussi C (2015). What do you feel if I apply transcranial electric stimulation? Safety, sensations and secondary induced effects. Clin. Neurophysiol..

[71] Lee MD, Wagenmakers E-J (2014). Bayesian Cognitive Modeling: A Practical Course.

[72] Stearman EJ, Durso FT (2016). Vigilance in a dynamic environment. J. Exp. Psychol. Appl..

[73] Epling SL, Edgar GK, Russell PN, Helton WS (2019). Is semantic vigilance impaired by narrative memory demands? Theory and applications. Hum. Factors J. Hum. Factors Ergon. Soc..

[74] Moray N, Haudegond S (1998). An absence of vigilance decrement in a complex dynamic task. Proc. Hum. Factors Ergon. Soc. Annu. Meet..

[75] Pop VL, Stearman EJ, Kazi S, Durso FT (2012). Using engagement to negate vigilance decrements in the NextGen environment. Int. J. Hum.-Comput. Interact..

[76] Luna FG, Tortajada M, Martín-Arévalo E, Botta F, Lupiáñez J (2022). A vigilance decrement comes along with an executive control decrement: Testing the resource-control theory. Psychon. Bull. Rev..

[77] Coulborn S, Bowman H, Miall RC, Fernández-Espejo D (2020). Effect of tDCS over the right inferior parietal lobule on mind-wandering propensity. Front. Hum. Neurosci..

[78] Filmer HL, Marcus LH, Dux PE (2021). Stimulating task unrelated thoughts: tDCS of prefrontal and parietal cortices leads to polarity specific increases in mind wandering. Neuropsychologia.

[79] Martínez-Pérez V (2023). Vigilance decrement and mind-wandering in sustained attention tasks: Two sides of the same coin?. Front. Neurosci..

[80] Figeys M, Loucks TM, Leung AWS, Kim ES (2023). Transcranial direct current stimulation over the right dorsolateral prefrontal cortex increases oxyhemoglobin concentration and cognitive performance dependent on cognitive load. Behav. Brain Res..

[81] Hsu W-Y, Zanto TP, Anguera JA, Lin Y-Y, Gazzaley A (2015). Delayed enhancement of multitasking performance: Effects of anodal transcranial direct current stimulation on the prefrontal cortex. Cortex.

[82] Vergallito A (2018). What is difficult for you can be easy for me. Effects of increasing individual task demand on prefrontal lateralization: A tDCS study. Neuropsychologia.

[83] Filmer HL, Mattingley JB, Dux PE (2013). Improved multitasking following prefrontal tDCS. Cortex.

[84] Weiss M, Lavidor M (2012). When less is more: Evidence for a facilitative cathodal tDCS effect in attentional abilities. J. Cogn. Neurosci..

[85] Miniussi C, Harris JA, Ruzzoli M (2013). Modelling non-invasive brain stimulation in cognitive neuroscience. Neurosci. Biobehav. Rev..

[86] Coffman BA (2012). Impact of tDCS on performance and learning of target detection: Interaction with stimulus characteristics and experimental design. Neuropsychologia.

[87] Falcone B, Coffman BA, Clark VP, Parasuraman R (2012). Transcranial direct current stimulation augments perceptual sensitivity and 24-hour retention in a complex threat detection task. PLoS ONE.

[88] Caggiano DM, Parasuraman R (2004). The role of memory representation in the vigilance decrement. Psychon. Bull. Rev..

[89] Thomson DR, Besner D, Smilek D (2013). In pursuit of off-task thought: Mind wandering-performance trade-offs while reading aloud and color naming. Front. Psychol..

[90] Krasich K (2018). Gaze-based signatures of mind wandering during real-world scene processing. J. Exp. Psychol. Gen..

[91] Mackie RR (1987). Vigilance research—Are we ready for countermeasures?. Hum. Factors J. Hum. Factors Ergon. Soc..

