# Supplementary material for: HD-tDCS mitigates the executive vigilance decrement only under high cognitive demands
Source: Sci Rep. 2024 Apr 3;14:7865. doi: 10.1038/s41598-024-57917-y (PMC10991279; doi:10.1038/s41598-024-57917-y)
Supplement: Supplementary file 1 — Supplementary Information. [file 41598_2024_57917_MOESM1_ESM.docx]

# **Supplementary Material**

## **Appendix A**. Blinding Efficacy

As total and individual subjective sensation data did not follow a normal distribution (*p* < .001 for Shapiro-Wilk test in all conditions), we performed a Mann-Whitney test to test for the blinding efficacy, followed up with a Bayesian Mann-Whitney test, showing that there is only anecdotal evidence for group differences in the total discomfort and pinching that was reported.

**Table A1**.

*Sensations between the anodal and sham HD-tDCS groups.*

| **Sensation** | **U** | **p** |  | **BF_10_** |
| --- | --- | --- | --- | --- |
| Total Discomfort | 2190 | 0.037 |  | 1.067 |
| Itching | 1968 | 0.336 |  | 0.28 |
| Pain | 1923 | 0.137 |  | 0.303 |
| Burning | 2001 | 0.170 |  | 0.292 |
| Warmth/Heat | 1883.5 | 0.574 |  | 0.241 |
| Pinching | 2166 | 0.001 |  | 0.926 |
| Metallic/Iron taste | 1801.5 | 0.989 |  | 0.255 |
| Fatigue | 1649 | 0.143 |  | 0.304 |

## **Appendix B.** Additional results for EV trials: single vs. dual load

**Single vs. Dual Load Conditions (*N* = 119 after behavioural filter)**

**False Alarms** (**FA**) for EV trials showed a significant decrement across Blocks, *F*(5, 575) = 18.73, *p* < .001, *ƞ_p_^2^* = .14. This effect did not interact with Stimulation Condition (*F* < 1), Task Type, *F*(5, 575) = 1.25, *p* = .284, *ƞ_p_^2^* = .01, or an interaction of both (*F* < 1).

The **Response Bias** (**B”**) index of EV trials showed a significant increment across Blocks, *F*(4.54, 521.90) = 29.92, *p* < .001, *ƞ_p_^2^* = .21, without any significant double or triple interaction with Stimulation Condition or Task Load (all *F*’s < 1).

## **Appendix C.** Additional results for EV trials: not triple vs triple load

**Not triple vs. Triple Load Conditions (*N* = 240 after behavioural filter)**

**False Alarms (FA**) showed a significant decrement across Blocks, *F*(4.65, 1097.98) = 33.01, *p* < .001, *ƞ_p_^2^* = .12. This effect did not interact with Stimulation Condition (*F* < 1), or Updated Task Type, *F*(4.65, 1097.99) = 1.55, *p* = .175, *ƞ_p_^2^* = .01, nor was there a significant interaction of both (*F* < 1).

The **Response Bias** (**B”**) index of EV trials, showed a significant increment across Blocks, *F*(4.57, 1078.67) = 44.03, *p* < .001, *ƞ_p_^2^* = .16, and the levels of Updated Task Type, *F*(1, 236) = 95.28, *p* < .001, *ƞ_p_^2^* = .29. This increment did not differ across Stimulation Conditions (*F* < 1). The triple Block × Stimulation Condition × Updated Task Type interaction was also not significant (*F* < 1). However, the Block × Updated Task Type interaction was significant, *F*(4.57, 1078.67) = 4.45, *p* = .006, *ƞ_p_^2^* = .01. While the overall B” was higher in the Triple (*M* = .51, *SD* = .53), than the Not Triple condition (*M* = -.17, *SD* = .56), the increment with time-on-task, was significantly less steep in the Triple condition compared to the Not-triple condition, *F*(1, 119) = 23.36, *p* < .001, *ƞ_p_^2^* = .16.

## **Appendix D**. Additional results for AV trials

### **Mean RT – Only Dual condition.**

In the dual task condition, no baseline (1^st^ Block) differences between Stimulation Groups in Mean RT for AV trials were observed, *F* < 1. No significant increment of Mean RT across Blocks was observed, *F*(3.41, 197.66) = 1.78, *p* = .145 (BF_01_ = 8.24). The interaction with Stimulation group was also not significant, *F* < 1 (BF_01_ = 51.59).

### **Mean RT – Dual vs. Triple condition.**

An ANOVA with Mean RT across Blocks as the dependent variable and Stimulation Group and Task Load (dual vs. triple) as between-subject factors, showed a significant increment of Mean RT across Blocks, *F*(3.39, 392.63) = 7.03, *p* < .001, *ƞ_p_^2^* = .06. However, interactions with Stimulation Condition, *F*(3.39, 392.63) = 1.01, *p* = .397, *ƞ_p_^2^* = .01 (BF_01_ = 63.29), or Task Load, *F*(3.38, 392.63) = 2.16, *p* = .084, *ƞ_p_^2^* = .02 (BF_01_ = 4.68), were not significant, nor was there a significant triple Block × Stimulation Condition × Task Load interaction *F* < 1 (BF_01_ = 277.16).

## **Appendix E.** Analyses for subjective fatigue data

For subjective fatigue (*n* = 180) we combined data from the present study and from Hemmerich et al.^13^. As for this data no specific analyses were pre-registered, we carried out the following exploratory analyses. First, an omnibus ANOVA was conducted with Fatigue Type (mental/physical) and Fatigue Moment (baseline/pre-task/post-task) as dependent variables, and Stimulation Group (anodal/sham) and Task Load (single/dual/triple) as between-participant factors. We followed up with post-hoc tests for significant interactions. Then, a post-task–pre-task change score was computed for both fatigue types, which were included in two further ANOVAs with Stimulation Group and Task Load as between-participant factors, and further planned comparisons (testing differences between Stimulation Group for each level of Task Load). Finally, we calculated Pearson correlation coefficients to relate EV and AV performance (Slope of Hits and Slope of SD of RT, respectively) to mental and physical fatigue.

## **Appendix F.** Exploratory analyses on Subjective Fatigue Measures and their relationship to HD-tDCS efficacy

The omnibus ANOVA revealed a main effect of Fatigue Type, *F*(1, 173) = 53.51, *p* < .001, *ƞ_p_^2^* = .24, as overall mental fatigue (*M* = 3.66, *SD* = 2.32) was higher than physical fatigue (*M* = 2.91, *SD* = 1.96). The significant main effect of Fatigue Moment, *F*(1.62, 280.43) = 185.85, *p* < .001, *ƞ_p_^2^* = .52, reflected higher overall fatigue levels in the post-task (*M* = 4.44, *SD* = 2.45), compared to baseline (*M* = 2.81, *SD* = 1.75) and pre-task assessments (*M* = 2.59, *SD* = 1.77). Refer to **Appendix G of the Supplementary Material** for a full table of results. The significant Fatigue Type × Fatigue Moment interaction, *F*(1.67, 280.43) = 56.37, *p* < .001, *ƞ_p_^2^* = .25, showed (via Tukey corrected post-hoc tests) that, without baseline differences between Fatigue Type, [*t*(178) = 2.43, *p* = .148; *M_mental_* = 2.97, *SD* = 1.89; *M_physical_* = 2.66, *SD* = 1.62], mental fatigue was incrementally higher than physical fatigue at pre-task [pre task: *t*(178) = 3.02, *p* = .003; *M_mental_* = 2.79, *SD* = 1.88, *M_physical_* = 2.41, *SD* = 1.65], and post-task [*t*(178) = 12.19, *p* < .001, *M_mental_* = 5.22, *SD* = 2.35, *M_physical_* = 3.67, *SD* = 2.33].

The Fatigue Moment × Stimulation Group × Task Load interaction was marginally significant, *F*(3.24, 280.43) = 2.47, *p* = .057, *ƞ_p_^2^* = .03. We explored this marginal triple interaction separately for each Fatigue Type, using the post-task–pre-task change, as baseline and pre-task fatigue did not differ significantly (both *p*’s > .321). The ANOVA on **physical fatigue change** revealed a significant Stimulation Group × Task Load interaction, *F*(2, 173) = 4.16, *p* = .017, *ƞ_p_^2^* = .05. Planned contrasts showed that physical fatigue increments for the dual task load were higher in the sham group (*M* = 1.87, *SD* = 1.81) as compared to the anodal group (*M* = 0.87, *SD* = 1.66), *t*(173) = 2.33, *p* = .021, as shown in **Fig. G.1.a** However, the single task condition showed a marginally significant opposite pattern, *t*(173) = -1.70, *p* = .090, with lower physical fatigue in the sham (*M* = 0.86, *SD* = 1.51) compared to the anodal group (*M* = 1.60, *SD* = 1.91). The triple task condition showed no significant differences between stimulation conditions (*t*(173) = -0.23, *p* = .816). Additionally, the ANOVA on the pre-post task change for **mental fatigue** showed no main effects nor interaction (all *F*’s < 1).

Note that, whilst prior analyses steps show some differences between load conditions, the tendency of all correlations between fatigue and performance follow the same pattern for each Task Load × Stimulation Condition combination. Although the most prominent results were observed in the dual task condition (see **Appendix H of the Supplementary Material** for further statistical details), which aligns with the previously observed results, given this overarching pattern, results are combined across Task Load levels to achieve a more adequate sample size for correlations. As can be seen in **Fig. G.1.b**, in the anodal groups a mitigated EV decrement (less negative Slope of Hits) was related to lower fatigue ratings [physical fatigue: *r*(90) = -.319, *p* = .002 (BF_10_ = 13.34); marginally for mental fatigue: *r*(90) = -.199, *p* = .060 (BF_10_ = 0.75)]; whereas in the sham groups fatigue measures were independent of EV performance [physical: *r*(89) = .079, *p* = .461 (BF_01_ = 5.77); mental: *r*(89) = .015, *p* = .887 (BF_01_ = 7.47)]. This same pattern was also observed for AV performance (see **Fig G.1.c**): a mitigated AV decrement (lower Slope of SD of RT) was related to lower fatigue ratings in the anodal groups [physical: *r*(60) = .386, *p* = .002 (BF_10_ = 14.80); mental: *r*(60) = .327, *p* = .011 (BF_10_ = 3.87)], which was not observed in the sham groups [physical: *r*(60) = -.073, *p* = .581 (BF_01_ = 5.34); mental: *r*(60) = .020, *p* = .879 (BF_01_ = 6.14)].


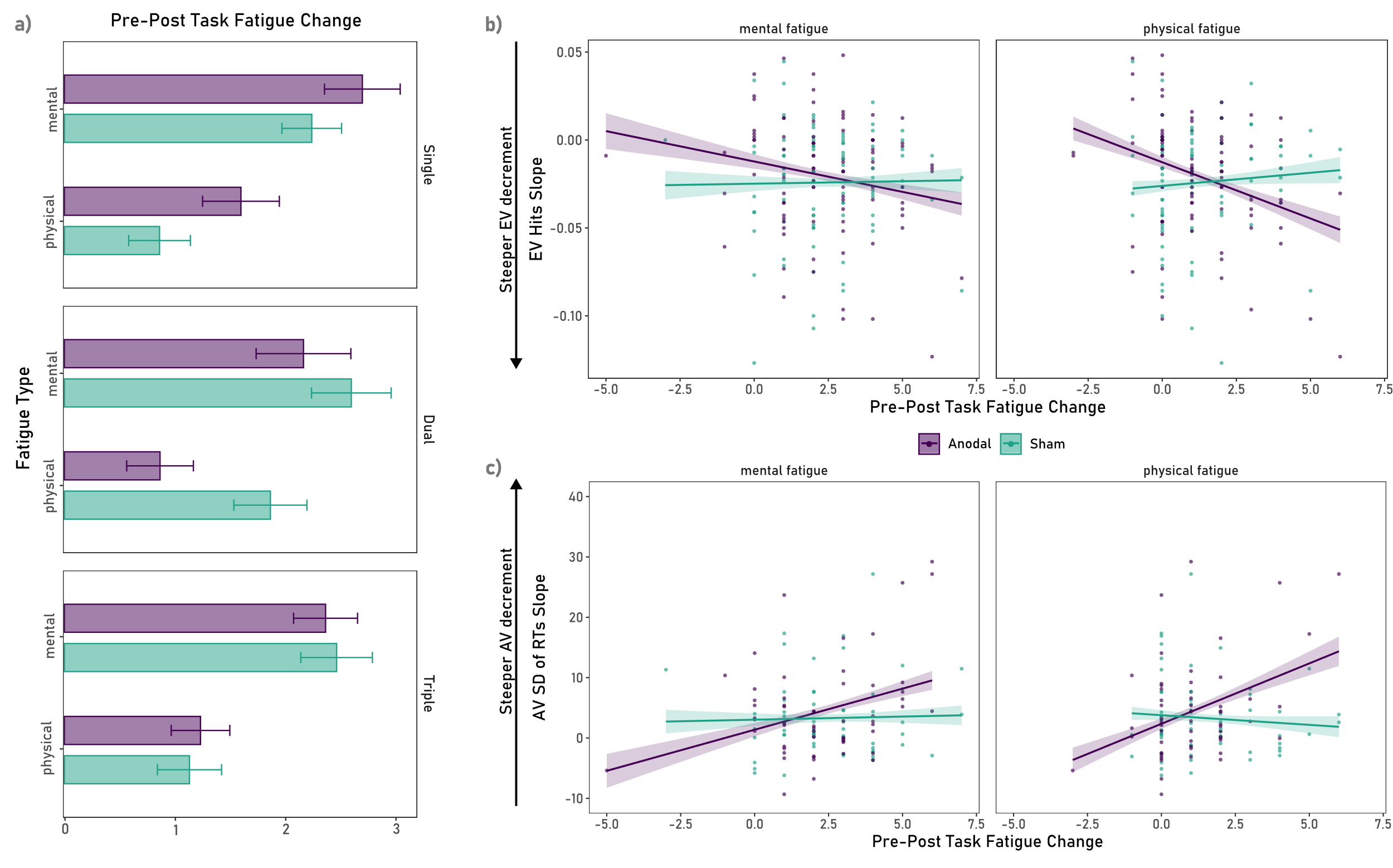


**Figure G.1.** Subjective Fatigue and its interaction with the EV and AV decrements. **a)** Mental and physical fatigue reported as a function of Task Load and Stimulation Group. **b)** Relationship between the Hits Slope (EV decrement) and the change in pre-post task fatigue for mental (left) and physical (right) fatigue. **c)** Relationship between SD of RT (AV decrement) and the change in pre-post task fatigue. Note that in this last plot: only the dual and triple task conditions are included (as no AV observations are included in the single task).

## **Appendix G.** Subjective fatigue Results

**Table E1**

*Mean (SD in grey) subjective fatigue by Fatigue Type, Fatigue Moment, Task Load and Stimulation Condition*

|  |  |  | **Single Task** | | | | **Dual Task** | | | | **Triple Task** | | | |
| --- | --- | --- | --- | --- | --- | --- | --- | --- | --- | --- | --- | --- | --- | --- |
| **Fatigue Moment** | **Fatigue Type** |  | **Sham** | | **Anodal** | | **Sham** | | **Anodal** | | **Sham** | | **Anodal** | |
| **Baseline** | Mental |  | 2.97 | 1.94 | 3.30 | 2.18 | 3.10 | 1.92 | 2.33 | 1.71 | 3.20 | 1.65 | 2.90 | 1.88 |
|  | Physical |  | 3.14 | 1.79 | 3.10 | 1.65 | 2.50 | 1.64 | 2.17 | 1.39 | 2.77 | 1.61 | 2.30 | 1.51 |
| **Pre-task** | Mental |  | 2.93 | 1.81 | 3.03 | 2.21 | 3.03 | 1.94 | 2.37 | 1.61 | 2.47 | 1.53 | 2.93 | 2.15 |
|  | Physical |  | 2.86 | 1.81 | 2.67 | 1.83 | 2.13 | 1.41 | 2.07 | 1.70 | 2.37 | 1.30 | 2.37 | 1.81 |
| **Post-task** | Mental |  | 5.17 | 2.22 | 5.73 | 1.87 | 5.63 | 2.67 | 4.53 | 2.56 | 4.93 | 2.26 | 5.30 | 2.44 |
|  | Physical |  | 3.72 | 2.69 | 4.27 | 1.86 | 4.00 | 2.52 | 2.93 | 2.27 | 3.50 | 2.01 | 3.60 | 2.49 |

## **Appendix H.** Correlations between subjective fatigue and the slope of the EV and AV decrements, presented individually for each experimental condition.

**Table F1.**

*EV Hits Slope and Mental/Physical Fatigue Change as a function of task and stimulation condition*

|  |  |  |  | EV Hits Slope –  Mental Fatigue Change | | |  | EV Hits Slope –  Physical Fatigue Change | | |
| --- | --- | --- | --- | --- | --- | --- | --- | --- | --- | --- |
|  |  | *N* |  | r | *p* | BF_10_ |  | r | *p* | BF_10_ |
| Single task | sham | 29 |  | -.302 | .111 | 0.78 |  | .092 | .637 | 0.257 |
|  | anodal | 30 |  | -.187 | .322 | 0.36 |  | -.315 | .090 | 0.895 |
| Dual task | sham | 30 |  | .307 | .099 | 0.83 |  | .157 | .408 | 0.315 |
|  | anodal | 30 |  | -.138 | .466 | 0.29 |  | -.26 | .165 | 0.569 |
| Triple task | sham | 30 |  | -.053 | .782 | 0.24 |  | .011 | .953 | 0.227 |
|  | anodal | 30 |  | -.287 | .125 | 0.70 |  | **-.374*** | **.042*** | **1.644** |

**Table E2.**

*AV SD of RT slope and Mental/Physical Fatigue Change as a function of task and stimulation condition*

|  |  |  |  |  | AV SD of RT Slope –  Mental Fatigue Change | | |  | AV SD of RT Slope –  Physical Fatigue Change | | |
| --- | --- | --- | --- | --- | --- | --- | --- | --- | --- | --- | --- |
|  |  | *N* |  |  | r | *p* | BF_10_ |  | r | *p* | BF_10_ |
| Dual task | sham | 30 |  |  | **-.327** | **.078** | 1.00 |  | -.257 | .171 | 0.556 |
|  | anodal | 30 |  |  | **.457*** | **.011*** | **4.91** |  | **.494**** | **.006**** | **9.949** |
| Triple task | sham | 30 |  |  | **.364*** | **.048*** | **1.47** |  | .135 | .478 | 0.289 |
|  | anodal | 30 |  |  | .159 | .402 | 0.32 |  | .260 | .165 | 0.571 |
